# Hidden hypertension and early cardiac alterations: insights from ambulatory blood pressure monitoring in children and adolescents

**DOI:** 10.1186/s13052-026-02319-1

**Published:** 2026-08-22

**Authors:** Mona Saber Mohamed Kamel, Sonia Ali El-Saiedi, Ayman Elsayed Shafei, Ashraf Abdel Baky Salama, Dalia Gaber Sos Boles, Mahmoud Othman Aboudeif

**Affiliations:** 1 Pediatric Department, Faculty of Medicine, Pediatric Cardiologist, Modern Technology and Information University, El-Hadaba El Wosta, 5th District, Mokattam, Cairo, Egypt; 2 Division of Pediatric Cardiology, Pediatrics Department, Faculty of Medicine, Cairo University, 2 Aly Basha Ibrahim, Mounira, Cairo, Egypt; 3 Sport and Physical Medicine, Faculty of Medicine, St. Petersburg University in Cairo, Wadi El Nile Medical Complex, Qubba, Cairo, Egypt; 4 Pediatric Medicine, Pediatric Department, Faculty of Medicine, Ain Shams University, El-Hadaba El Wosta, 5th District, Mokattam, Cairo, Egypt; 5 Community Medicine Department, Faculty of Medicine, Ain Shams University, Al-Khalifa El-Ma’moun St, Abbassia, Cairo, Egypt; 6 Division of Pediatric Cardiology, Pediatric Cardiology, Pediatrics Department, Cairo University, 2 Aly Basha Ibrahim. Mounira, Cairo, Egypt

**Keywords:** Hypertension, Elevated blood pressure, Schoolchildren, Adolescents, Ambulatory blood pressure, Lifestyle, Obesity

## Abstract

**Background:**

Hypertension is increasingly recognized as a significant health concern among children and adolescents, often associated with obesity and lifestyle choices. Early-onset hypertension can elevate the risk of cardiovascular diseases in adulthood, making early detection crucial. As it is often asymptomatic, routine screening is essential. Schools serve an effective environment for identifying at-risk students. Accurate diagnosis requires standardized protocols because blood pressure (BP) can vary based on age, sex, and height. Addressing pediatric hypertension is key to reducing long-term cardiovascular morbidity and mortality.

**Methods:**

The study included 354 students: 234 primary grade 3 students (8–9 years) and 120 secondary grade 9 students (~ 15 years). Assessments included medical history, physical examination (weight, height, BMI, and BP). BP was measured using standardized methods and classified by established percentiles. Ambulatory Blood Pressure Monitoring (ABPM) was performed for students with elevated BP to analyze 24-hour readings, dipping status, and pulse pressure, and to classify hypertension. Echocardiography was performed on all students with elevated BP.

**Results:**

Significant differences were observed in BMI and hypertension prevalence between Grade 3 and Grade 9. In Grade 3, 45.7% were underweight, 31.9% had normal BMI, and 22% were overweight or obese; in Grade 9, 81.5% were overweight or obese. Clinic-based hypertension was higher in Grade 3 (15.4%) compared to Grade 9 (12.5%), sustained hypertension was confirmed by ABPM to rule out white-coat hypertension, results correlated with BMI Z scores and lifestyle. Despite being asymptomatic, cardiac changes were observed: the mean interventricular septal thickness (IVSd) was 0.75 cm in Grade 3 versus 1.07 cm in Grade 9.

**Conclusions:**

ABPM is crucial for accurate diagnosis of hypertension in schoolchildren and adolescents, clarifying the relationship between obesity, lifestyle, and elevated BP. The study underscores that early cardiac remodeling was already present in asymptomatic children, emphasizing the need for early interventions, including weight management and lifestyle modifications, to reduce hypertension and future cardiovascular risk.

## One-sentence summary

This study delivers one of the first comprehensive community-based assessments of pediatric blood pressure in our region, revealing a high burden of hidden hypertension and early cardiovascular alterations, and underscoring the urgent need for routine screening as obesity-related health risks continue to rise among children and adolescents.

## Introduction

Hypertension, or elevated blood pressure (BP), is no longer considered solely a condition viewed as a condition exclusive to adults. The rising prevalence of hypertension among children and adolescents has become a significant public health concern [1]. Early onset of hypertension during childhood is associated with a higher likelihood of developing cardiovascular diseases (CVD) and related complications in adulthood [2]. Screening for hypertension in school-aged children and adolescents is critical for early detection, timely intervention and prevention of long-term health consequences [3].

The global burden of pediatric hypertension is rising, paralleling the increasing prevalence of childhood obesity and other lifestyle-related risk factors [4]. Studies have reported that the prevalence of hypertension in children varies between 1% and 5% in normotensive populations, but it increases significantly in populations with risk factors such as obesity, sedentary lifestyles, and poor dietary habits [5]. Moreover, a large proportion of children with elevated BP remain undiagnosed due to the asymptomatic nature of the condition, emphasizing the need for routine screening in this population [6].

Hypertension in children often serves as an early indicator of broader health issues, including obesity, metabolic syndrome and renal or cardiovascular dysfunction. Research demonstrates that elevated BP in childhood frequently tracks into adulthood leading to early onset of conditions such as coronary artery disease, stroke, and heart failure [7]. Identifying and managing elevated BP during childhood provides an opportunity to disrupt this progression and potentially reducing the long-term burden of cardiovascular morbidity and mortality [8].

However, diagnosing hypertension in children presents unique challenges. BP levels in this age group can vary significantly based on factors like age, sex, and height, necessitating the use of percentile-based reference charts for accurate assessment [4]. Furthermore, phenomena such as white coat hypertension and transient BP fluctuations caused by anxiety or physical activity can complicate accurate measurement, highlighting the need for standardized protocols and multiple readings to confirm diagnoses [9].

Schools offer an accessible and practical setting for hypertension screening programs, facilitating the early identification of at-risk children on a large scale. School-based screening initiatives have shown promise in detecting previously unrecognized cases of elevated BP while also fostering awareness of healthy lifestyle habits among students and their families. Such programs can play a pivotal role in addressing the rising prevalence of hypertension and its associated risk factors in young populations [10].

The increasing prevalence of pediatric hypertension emphasizes the importance of proactive screening and preventive measures. By implementing school-based BP screening programs, healthcare professionals can identify at-risk children early and initiate timely interventions. These efforts hold the potential to mitigate the future burden of hypertension-related complications, improving overall public health outcomes [11, 12].

The current study aims to screen blood pressure among school children and to conduct 24-hour ambulatory blood pressure monitoring (ABPM) for those identified with elevated blood pressure levels.

## Methodology

A School-based cross-sectional observational study was conducted at Primary and Secondary private Schools in Egypt, involving a total of 354 students divided into two groups: **Group 1**: 234 primary school students in grade 3, aged 8–9 years. **Group 2**: 120 secondary school adolescents in grade 9, aged around 15 years. **Inclusion criteria**: All students were included in the study. **Exclusion criteria**: students with known hypertension, renal endocrinal, cardiac disease or those taking medications that alter blood pressure. One primary school student, whose blood pressure was elevated secondary to Kawasaki disease, we ensured that the student was under regular follow-up with her physician and confirmed that she was adhering to the recommended treatment protocol, but excluded from our statistical analysis.

**Study Procedures** The students underwent comprehensive assessments, including medical history, physical examination, blood pressure (BP) measurement, and additional diagnostic procedures. **A)-Complete Medical History** focused on Personal History, History of medications or conditions affecting BP. Family History of Hypertension or cardiovascular disease. Dietary Habits: (salt intake, fast food and processed food) categorized as poor dietary habits. Physical Activity History: Defined as sufficient (≥ 3 sessions/week, each lasting ≥ 1 h of intense exercise) or insufficient (< 3 sessions/week) [13, 14]. **B)- Physical Examination** included measurements of weight, height, and body mass index (BMI). BMI was calculated as weight (kg) divided by height squared (m²) [15, 16]. Nutritional status was classified using age- and sex-specific BMI forage Z-scores based on the World Health Organization (WHO) growth reference (2007) for children and adolescents: severe thinness (Z-score < -3), moderate thinness (Z-score ≥ -3 to < -2), mild thinness (Z-score ≥ -2 to < -1), normal (Z-score ≥ -1 to ≤ + 1), overweight (Z-score > + 1 to ≤ + 2), obese class I (Z-score > + 2 to ≤ + 3), obese class II (Z-score > + 3 to ≤ + 4), and obese class III (Z-score > + 4). Pediatric BMI Z-scores were used to ensure accurate age- and sex-specific assessment of nutritional status in both grade 3 and grade 9 students [17, 18] **C) Blood Pressure Measurement**: Performed using standard and oscillometric sphygmomanometers adhering to specific protocols: Students were seated in a quiet room for 3–5 min before measurement was measured on the right arm for consistency with the arm at heart level. Appropriate cuff size was used (bladder length 80–100% of the arm circumference; width ≥ 40%). Auscultatory BP was measured using a stethoscope with SBP and DBP determined by Korotkoff sounds [4]. **D)** BP was classified based on the following criteria: **Normal BP**: SBP and DBP < 90th percentile (based on age, sex, and height). **Prehypertension**: SBP and/or DBP ≥ 90th and < 95th percentile. **Confirmed Hypertension (HTN)**: SBP and/or DBP ≥ 95th percentile, further classified into stages 1 and 2 [4, 19]. **E) Ambulatory Blood Pressure Monitoring (ABPM)**: ABPM was conducted for students with elevated BP (SBP ≥ 90 percentile): Non-invasive, 24-hour monitoring using Meditech Ambulatory BP and ECG monitors. Measurements were taken every 30 min for 24 h. Data was considered acceptable if at least 70% of readings were successful over a minimum of 18 h or 40 readings in a 24-hour period [4]. Parameters included: Mean Arterial Pressure (MAP), 24-Hour Pulse Pressure, Dipping Status. Students were categorized as having normal BP, white coat hypertension, prehypertension or confirmed hypertension based on BP percentiles for age, sex and height [20, 21]. **F) Echocardiographic Assessment**: for students with elevated BP (systolic Z score ≥ 90). Echocardiography was performed not only to assess cardiac function and early hypertrophy but also to exclude structural causes of elevated blood pressure, with measurements conducted as part of broader school cardiovascular screenings to minimize observer bias and ensure standardized and objective assessments. Transthoracic echocardiography was performed to assess LV dimensions, wall thickness, and function. Interventricular septal and posterior wall thickness, as well as ejection fraction, were measured by a trained pediatric cardiologist. Results were indexed and correlated with age- and sex-specific Z-scores.

### Statistical analysis

Data was analyzed using the Statistical Package for Social Sciences (SPSS version 25). The Kolmogorov-Smirnov test was used to assess normality for continuous variables. Descriptive analyses were performed to obtain the means, and deviations for quantitative data Numbers and frequencies for qualitative data. Different types of graphs were used according to the type and distribution of data (bar, pie and histogram plot), Bivariate analyses were performed using the independent samples t test, and the chi-squared test for categorical variables. P value < 0.05 was considered significant.

## Results

The present study provides a comprehensive assessment of the anthropometric and health-related characteristics of students in Grades 3 and 9, highlighting age-related patterns and differences across key nutritional and clinical indicators. As shown in Table 1, the two groups differed in their growth profiles, reflecting the natural progression from childhood to adolescence. With the older group had higher body measurements across all parameters. Correspondingly, the mean BMI Z-score increased from 1.21 ± 0.72 in Grade 3 to 2.54 ± 0.68 in Grade 9, reflecting a clear shift toward overweight and obesity with age. This standardized assessment highlights that the observed increase cannot be attributed to normal growth alone but indicates a substantial rise in excess adiposity during adolescence.

**Table 1:** Demographic distribution and anthropometric measures among both studied groups

		Grade 3 *N* = 234	Grade 9 *N* = 120
Age	Mean (SD)	8.5	14
	Min- Max	8–9	13–15
Sex	Female	105 (55.3%)	54 (45%)
	Male	130 (44.6%)	66 (55%)
Weight	Mean (SD)	32.76 (9.27)	59.77 (14.23)
	Min- Max	20–59	37–115
Hight	Mean (SD)	125.90 (10.46)	144.18(11.96)
	Min- Max	130–143	140–176
Hight Z score	Mean (SD)	-0.70- (1.22)	
	Min- Max	-3.50, 3.39	
BMI	Mean (SD)	20.56(5.43)	29.88 (6.85)
	Min- Max	13.32–45.40	18.4- 59.52
BMI Z score	Mean (SD)	1.28(1.34)	1.86(1.01)
	Min- Max	-3.87- 3.32.	-0.35- 3.99.
Waist	Mean (SD)	66 (9)	72(10)
	Min- Max	33–98	57–105
Hip	Mean (SD)	76(8)	93(13)
	Min- Max	54–102	45–137
waist/hip	Mean (SD)	0.87(0.12)	0.79 (0.15)
	Min- Max	0.50–1.44	0.59- 2.00

Building on these anthropometric findings, Table 2 presents the distribution of student’s nutritional status based on WHO BMI-for-age Z-scores. In Grade 3 (*N* = 234), undernutrition was uncommon, with severe, moderate, and mild thinness observed in 2.1%, 6.0%, and 9.8% of students, respectively, while 33.3% had normal weight. Overweight and obesity were already present, affecting 21.4% (overweight) and 27.3% (obese classes I–III) of students. In, Grade 9 students (*N* = 120) showed a marked shift toward excess adiposity, with only 11.7% in the normal range, 30.8% overweight, and 56.6% obese (classes I–II). This trend highlights a clear transition to overweight and obesity in adolescence.

**Table 2:** Distribution of nutritional status according to WHO BMI-for-age Z-scores among both studied groups

Nutritional classification according to WHO BMI-for-age Z-scores	Grade 3 *N* = 234	%	Grade 9 *N* = 120	%
Severe thinness	5	2.1%	0	0.0%
Moderate thinness	14	6.0%	0	0.0%
Mild thinness	23	9.8%	1	0.8%
Normal	78	33.3%	14	11.7%
Overweight	50	21.4%	37	30.8%
Obese class I	45	19.2%	43	35.8%
Obese class II	15	6.4%	25	20.8%
Obese class III	4	1.7%	0	0.0%

**Fig. 1:**
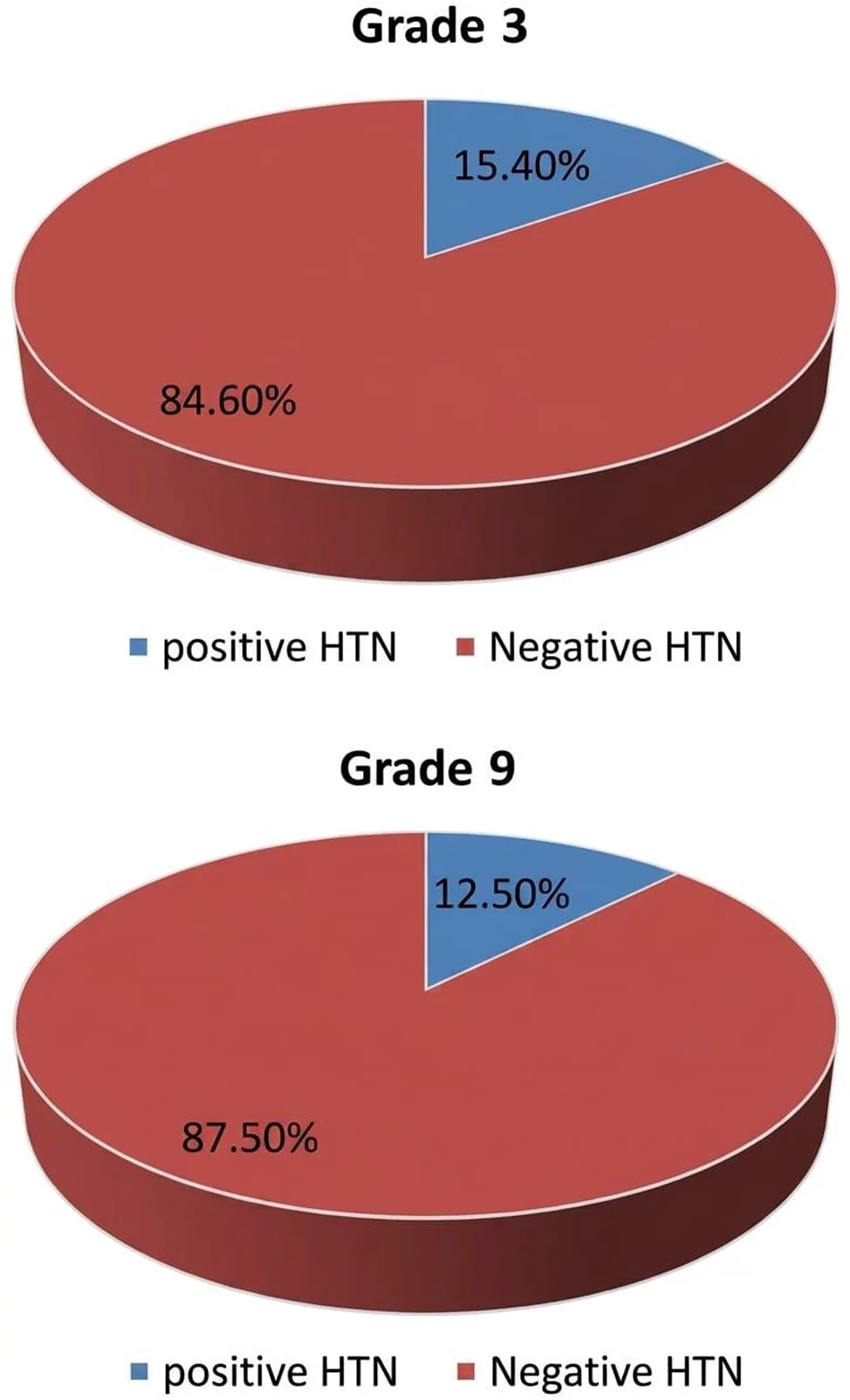
Pie chart showing the distribution of students with normal and elevated blood pressure in Grades 3 and 9. The numbers (N) and percentages (%) of students in each category (elevated BP and normal BP) are indicated. Grade 3: 36/234 (15.4%) with elevated BP, 198/234 (84.6%) normal. Grade 9: 15/120 (12.5%) with elevated BP, 105/120 (87.5%) normal

In light of these pronounced shifts in nutritional status, the analysis also examined blood pressure patterns to determine whether similar age-related differences were evident. The pie chart in Fig. 1, shows that hypertension was detected in 15.4% (36 /234) of Grade 3 students and 12.5% (15/120) of Grade 9 students, while the majority in both grades, 84.6% and 87.5%, respectively tested negative.

To further investigate the relationship between body composition and blood pressure, Table 3, compares anthropometric parameters between hypertensive and normotensive students in Grades 3 (*N* = 234) and 9 (*N* = 120). In Grade 3, hypertensive students had higher weight (46.29 vs. 30.27 kg, *P* < 0.001), BMI Z-score (2.54 ± 0.68 vs. 1.21 ± 0.72, *P* < 0.001) and waist (74 vs. 64 cm, *P* < 0.001). In Grade 9, hypertensive students had higher weight (81.80 vs. 56.56 kg, *P* < 0.001), BMI Z-score (3.21 ± 0.68 vs. 1.93 ± 0.78, *P* < 0.001) waist (84 vs. 71 cm, *P* < 0.001), and hip (107 vs. 91 cm, *P* < 0.001). Lifestyle factors were also linked to hypertension as shown in Table 4 students doing sports ≥ 3 times/week had lower HTN prevalence (*P* < 0.001), while frequent beverage consumption (4–6 times/week) and junk food intake were associated with higher HTN (beverages: *P* < 0.001; junk food: Grade 3 *P* = 0.002, Grade 9 *P* = 0.005).

**Table 3:** Comparison of anthropometric measurements between hypertensive students (Yes) and normal Blood pressure group (No) in both grades

	Grade 3 *N* = 234					Grade 9 *N* = 120				
	Prevalence of HTN z score					Prevalence of HTN z score				
	No*N* = 198		Yes*N* = 36		*P* valuet-test	NO*N* = 105		Yes*N* = 15		*P* value t-test
	Mean	SD	Mean	SD		Mean	SD	Mean	SD	
Sex N (%)										
Female Male	89 (44.9%) 109(55.1%)		16(44.4%) 20 (55.6%)		X^2^ =0.955	43 (41%) 62(59%)		10(66.7%) 5(33.3%)		X^2^ =0.610
Age	8.3	0.7	8.4	0.5	0.705	14.4	0.6	14.4	0.5	0.803
Wt	30.27	7.18	46.29	7.54	0.143	56.56	10.34	81.80	17.83	< 0.001*
Ht	125.47	11.07	128.25	5.58	0.02*	144.55	12.04	141.60	11.41	0.364
Z score	-0.79	1.18	-0.27	1.36	< 0.001*	.	.	.	.	
BMI	19.15	4.40	28.19	3.94	< 0.001*	28.14	4.98	41.90	5.98	< 0.001*
BMI Z score	1.21	0.72	2.54	0.68	< 0.001*	1.93	0.78	3.21	0.68	< 0.001*
Waist	64	7	74	10	< 0.001*	71	8	84	11	< 0.001*
Hip	74	7	83	10	0.075	91	11	107	12	< 0.001*
waist/hip	0.86	0.12	0.90	0.13	0.098	0.79	0.16	0.79	0.04	0.955

*sig p-value

**Table 4:** Association between lifestyle factors (sport, beverages, and junk food consumption) and prevalence of hypertension (HTN) Z-scores among grade 3 and 9 students

		Grade									
		3 *N* = 234					9 *N* = 120				
		prevalence of HTN z score					Prevalence of HTN z score				
		No *N* = 198		yes *N* = 36		Chi-squared test p-value	NO *N* = 105		Yes *N* = 15		Chi-squared test p-value
		N	%	N	%		N	%	N	%	
Sport	=<3 times /week	29	14.6%	28	77.8%	< 0.001	76	72.3%	15	100.0%	0.005*
	≥ 3times/week	169	85.4%	8	22.2%		29	27.6%	0	0.0%	
Beverages	Never	48	24.5%	3	8.3%	< 0.001*	14	13.7%	0	0.0%	< 0.001*
	1–3 times per week	139	70.9%	16	44.4%		85	83.3%	5	33.3%	
	4–6 times per week	9	4.6%	14	38.9%		2	2.0%	9	60.0%	
	Daily	0	0.0%	3	8.3%		1	1.0%	1	6.7%	
Junk food	Never	4	2.0%	0	0.0%	0.002*	1	1.0%	0	0.0%	0.005*
	1–3 times per week	189	95.9%	31	86.1%		100	97.1%	12	80.0%	
	4–6 times per week	4	2.0%	5	13.9%		2	1.9%	3	20.0%	
	Daily	0	0.0%	0	0.0%		0	0.0%	0	0.0%	

*sig p value

**Fig. 2:**
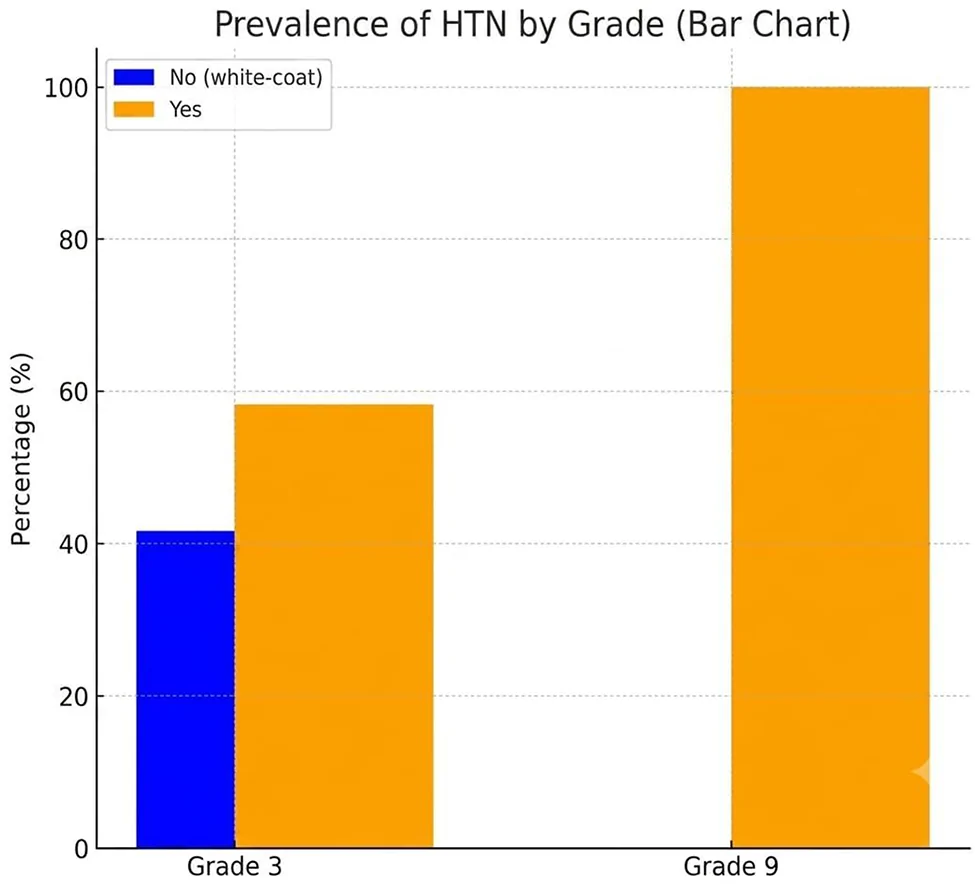
Bar chart illustrating the prevalence of white-coat hypertension (WCH), pre-hypertension, and confirmed hypertension in Grades 3 and 9 based on ambulatory blood pressure monitoring (ABPM) Z-scores. The numbers (N) and percentages (%) for each group are indicated. Grade 3: 15/36 (41.7%) with WCH, 12/36 (33.3%) pre-hypertensive, 9/36 (25%) hypertensive. Grade 9: 0/15 (0%) with WCH, 10/15 (66.7%) pre-hypertensive, 5/15 (33.3%) hypertensive

The analysis was then refined to examine the prevalence of white coat hypertension (WCH) using ambulatory BP Z-scores, providing insight into transient versus sustained hypertension. The Bar chart in Fig. 2, shows the percentages of “No (white coat)” and “Yes” for Grades 3 and 9 based on ambulatory BP Z-scores. In Grade 3 (*n* = 36), 15 students (41.7%) had WCH and 21 students (58.3%) had confirmed hypertension. In Grade 9 (*n* = 15), none of the students (0%) had WCH while the 15 students (100%) had confirmed hypertension.

Following the assessment of white coat hypertension (WCH), anthropometric measurements among Grade 3 students Table 5a, were compared across categories classified as white coat, pre-hypertensive, and hypertensive using Z-scores. Weight and BMI differed significantly (*p* < 0.001), with hypertensive students having the highest mean weight (55.50 ± 3.11 kg), BMI (32.69 ± 0.67) and BMI Z-score (2.36 ± 0.28) followed by pre-hypertensive (48.85 ± 4.81 kg, 29.62 ± 2.43 BMI Z-score 1.89 ± 0.32) and white coat students (40.93 ± 7.11 kg, 25.37 ± 3.90, BMI Z-score 1.12 ± 0.54), indicating a graded association between increased adiposity and blood pressure.

**Table 5a:** Comparison of anthropometric measurements across white coat, pre-hypertension, and hypertension categories among grade 3 students

	white coat *N* = 15		pre HTN *N* = 12		HTN *N* = 9		ANOVA *p*-value
	Mean	SD	Mean	SD	Mean	SD	
Wt	40.93	7.11	48.85	4.81	55.50	3.11	< 0.001*
Ht	127.00	6.49	128.82	4.95	130.50	4.36	0.466
Z score	− 0.58-	1.17	− 0.29-	1.35	0.99	1.71	0.120
BMI	25.37	3.90	29.62	2.43	32.69	0.67	< 0.001*
BMI Z score	1.12	0.54	1.89	0.32	2.36	0.28	< 0.001*
Waist	72	9	76	11	72	8	0.552
Hip	80	10	83	10	88	6	0.329
waist/hip	0.91	0.16	0.91	0.11	0.82	0.04	0.370

**Table 5b:** Comparison of anthropometric measurements between pre-hypertension and hypertension categories among grade 9 students

	pre HTN *N* = 10		HTN *N* = 5		t-test *p* value
	Mean	SD	Mean	SD	
Wt	81.80	19.34	81.80	16.45	0.99
Ht	145.20	9.74	134.40	12.03	0.083
BMI	41.25	7.13	43.20	2.75	0.571
BMI Zscore	2.76	0.68	3.12	0.68	0.312
Waist	86	10	81	13	0.434
Hip	109	11	102	13	0.344
waist/hip	0.79	0.04	0.79	0.03	0.956

A similar analysis was conducted among Grade 9 students in Table 5b, comparing pre-hypertensive and hypertensive groups. No statistically significant differences were observed in weight, BMI, or BMI Z-score (*P* > 0.05). BMI Z-scores were elevated in both groups (2.76 ± 0.68 vs. 3.12 ± 0.68, *P* = 0.312), indicating that excess adiposity was prevalent regardless of blood pressure category. This may reflect the uniformly high obesity burden in adolescents and the small sample size within this subgroup.

To further explore the impact of elevated blood pressure on the heart, Table 6a compares cardiovascular parameters among Grade 3 students classified as white coat, pre-hypertensive, and hypertensive. The hypertensive group had significantly higher SBP and DBP (morning and night), BP centiles, MAP, and high BP readings (*p* < 0.05), while pulse pressure and nighttime MAP were similar. Cardiac structural measurements, including interventricular septal thickness (IVSd) and left ventricular posterior wall thickness (LVPWd) with Z-scores, were elevated, indicating early left ventricular remodeling and hypertrophy associated with elevated blood pressure (*p* < 0.001).

**Table 6a:** Comparison of blood pressure parameters using 24 h ambulatory blood pressure monitoring and, echocardiographic measures across white coat, pre-hypertension, and hypertension categories among grade 3 students

	z score white coat grade 3						ANOVA *p* value
	white coat *N* = 15		pre HTN *N* = 17		HTN *N* = 4		
	Mean	SD	Mean	SD	Mean	SD	
PP overall	76	4	78	4	81	2	0.104
SBP max limit morn	105	8	115	4	124	1	< 0.001*
DBP max limit value morning	63	7	72	3	77	2	< 0.001*
SBP morning centile for age	59	18	93	4	99	0	< 0.001*
DBP morning centile for age	55	14	92	3	98	2	< 0.001*
SBP max limit value night	97	5	101	6	105	2	0.02*
Centile	61	19	90	11	95	0	< 0.001*
DBP max limit value night	59	3	63	5	67	1	0.003*
Centile	58	17	84	17	95	0	< 0.001*
MAP average daytime	82	4	86	6	89	3	0.011*
MAP average night time	70	4	74	6	74	6	0.080
SBP high readings	4	3	9	2	13	2	< 0.001*
DBP high readings	1	1	3	1	5	1	< 0.001*
FS	42	4	36	3	37	1	< 0.080
IVSd	0.75	0.14	0.95	0.07	1.07	0.08	< 0.001*
LVPWd	0.87	0.16	1.12	0.17	1.23	0.04	< 0.001*
Z score	1.38	1.14	3.11	0.74	3.82	0.32	< 0.001*

*sig p value

**Table 6b:** Comparison of blood pressure parameters using 24-hour ambulatory blood pressure monitoring and echocardiographic measures between pre-hypertension and hypertension categories among grade 9 students

	pre HTN *N* = 10		HTN *N* = 5		T-test *p* value
	Mean	SD	Mean	SD	
PP overall	83	5	83	7	0.877
SBP max limit morn	134	5	134	5	0.946
DBP max limit value morning	84	4	88	8	0.260
SBP morning centile for age	92	3	97	2	0.010*
DBP morning centile for age	92	4	96	2	0.146
SBP max limit value night	123	5	121	5	0.533
Centile	92	3	96	2	0.012*
DBP max limit value night	74	4	74	3	---
Centile	92	3	95	3	0.089
MAP average daytime	98	7	101	7	0.511
MAP average night time	82	8	83	4	0683
SBP high readings	9	1	11	1	0.005*
DBP high readings	6	2	5	1	0.428
FS	38	3	36	3	0.252
IVSd	1.16	0.14	1.14	0.07	0.775
LVPWd	1.28	0.09	1.52	0.15	0.012*
Z score	3.21	0.72	4.08	1.13	0.091

*sig p value

To examine whether the patterns observed in younger children persist into adolescence in Table 6b a similar analysis was conducted to compare cardiovascular parameters between pre-hypertensive and hypertensive Grade 9 students. The hypertensive group showed significantly higher systolic blood pressure (SBP) centiles morning (*p* = 0.010), night (*p* = 0.012), and high readings (*p* = 0.005) as well as increased LVPWd thickness (LVPWd, *p* = 0.012), indicating early structural cardiac changes associated with elevated systolic pressure.

## Discussion

The present study assessed blood pressure among school-aged children and adolescents, including 235 children in Grade 3 and 120 adolescents in Grade 9. In Grade 3, nutritional assessment using WHO BMI-for-age Z-scores demonstrated a dual burden of malnutrition. Thinness (severe, moderate, and mild combined) was observed in 17.9% of children, whereas 48.7% were classified as overweight or obese, and only 33.3% fell within the normal BMI range. These findings indicate that excess adiposity is already prevalent in younger children and exceeds the proportion of undernutrition.

In Grade 9, the pattern shifted markedly toward adiposity, with 30.8% categorized as overweight and 56.6% as obese, while only 11.7% had normal BMI-for-age and thinness was nearly absent (0.8%). The redistribution observed after applying BMI-for-age Z-scores reflects the methodological difference between adult BMI cut-offs and pediatric growth references, as Z-scores account for age- and sex-specific growth patterns, reclassifying some children into higher adiposity categories [37]. This approach provides a more accurate and age-appropriate assessment of nutritional status in children and adolescents [17].

The pronounced increase in overweight and obesity from childhood to adolescence highlights age-related lifestyle and socio-environmental transitions, rather than a rapid longitudinal change within individuals, given the cross-sectional design. During adolescence, increased autonomy in dietary choices, higher consumption of energy-dense processed foods, and reduced parental oversight contribute to excess caloric intake [1, 37]. Consistent with this, our cohort demonstrated a significant association between low physical activity (≤ 3 sessions/week) and elevated blood pressure, while frequent consumption of sugar-sweetened beverages and junk food was similarly linked to higher blood pressure, particularly among older students (Grade 9) [13, 29].

These findings indicate that the progression from childhood to adolescence is accompanied by behavioral patterns contributing to a positive energy balance and adiposity accumulation [13]. Reduced physical activity, increased screen time, and social eating behaviors collectively exacerbate weight gain during adolescence [14]. Similar age-related increases in overweight and obesity prevalence have been reported in regional studies in Egypt and the UAE, underscoring the public health relevance of early lifestyle interventions in this population [22, 37].

These findings align with research conducted in the United Arab Emirates (UAE) and Dubai, which also reported a continual rise in childhood and adolescent overweight and obesity. In Ras Al-Khaimah, a school survey of 44,942 students revealed increasing obesity rates, particularly among boys, whose extreme obesity rates were nine times higher than those of girls. Similarly, a Dubai school study identified 25.3% of adolescents as overweight and 15% as obese, attributing these patterns to unhealthy eating habits, including low consumption of fruit and vegetable intake and frequent fast-food intake [22, 23].

Blood pressure measurement in the present study further revealed that 15.4% of grade 3 students and 12.5% of grade 9 students had elevated blood pressure which could indicate early hypertension. In both grade 3 and grade 9, a significant association was found between elevated blood pressure and higher BMI Z score, waist circumference, and hip circumference reinforcing the link between obesity and hypertension.

The results of this study align with findings from Pollack et al. in northern Colorado, which demonstrated a clear association between higher BMI and hypertension in school-aged children [24] as well as Ejike et al. who demonstrated a strong link between central adiposity and increased blood pressure among adolescents in both urban and semi-urban settings. Together, these studies emphasize the need to address obesity and hypertension early to reduce cardiovascular risk [25].

To ensure accurate diagnosis, ambulatory blood pressure monitoring (ABPM) was utilized for students with elevated blood pressure. ABPM is considered the gold standard for diagnosing hypertension, providing a comprehensive 24-hour blood pressure profile that helps distinguish between white-coat hypertension (WCH), masked hypertension, and sustained hypertension, it also offers insights into nocturnal dipping which is a key predictor of cardiovascular risk [4]. The American Academy of Pediatrics (AAP) and the European Society of Hypertension recommend ABPM for confirming hypertension and tailoring management strategies [26, 27].

Among the 36 grade 3 students with elevated blood pressure, ABPM confirmed that 15 students had white-coat hypertension, 12 were classified with pre-hypertension, and 9 were diagnosed with sustained hypertension. These results highlight ABPM’s value in distinguishing transient from persistent hypertension elevations, and supporting clinical decision-making [28]. This study found a significant association between higher BMI Z score and sustained hypertension. Importantly, students with sustained hypertension had significantly higher BMI Z-scores compared to those with WCH (mean BMI Z-score 2.36 vs. 1.12), emphasizing that excess adiposity is a major contributor to elevated blood pressure in this population [4].

Several studies support using ABPM to confirm hypertension in children and adolescents. Sorof et al. reported that 25% of children first labeled hypertensive in clinic were reclassified as having WCH after ABPM. His study also showed that obesity is a major hypertension risk factor, with obese children displaying greater blood pressure variability than non-obese peers [29]. In contrast, our study did not observe this variability during daytime or sleep, indicating a different hypertension pattern in our cohort.

Our study also identified a positive link between carbonated drinks consumption and elevated blood pressure in grade 3 students. Children with sustained hypertension consumed substantially more carbonated beverages than non-hypertensive peers, indicating that high sugar-sweetened beverages intake may raise blood pressure. This finding is consistent with Nguyen et al., who reported a positive relationship between sugary drink consumption and increased blood pressure in children, independent of BMI [30, 31].

Among grade 9 students, the 15 adolescents with elevated blood pressure underwent ABPM interestingly, no cases of white-coat hypertension (WCH) were observed in Grade 9. This may be attributed to the small sample size (*n* = 15) and the orientation sessions conducted for students and families, which reduced anxiety. Furthermore, both the pre-hypertensive and hypertensive groups exhibited significantly elevated BMI Z-scores (2.76–3.12), reinforcing the role of obesity as a major risk factor for sustained hypertension.

Ten out of the 15 hypertensive adolescents were females, with five of them having sustained hypertension. Differences between boys and girls, particularly in fat distribution, may influence blood-pressure trajectories during adolescence, highlighting the need for future sex-specific analyses [32]. This observation reinforces the strong link between obesity, central adiposity, and elevated blood pressure, particularly among girls, as reported by Glonti et al. [33, 34], whereas hypertensive boys were fewer and predominantly within overweight or class I obesity ranges.

Of the hypertensive female students, four were in the premenstrual phase, three of whom fell within the elevated blood pressure group. Given the small number of students in this phase, conclusions regarding hormonal influences on blood pressure should be interpreted with caution. While hormonal fluctuations during puberty may contribute to BP variations, the primary contributors in this cohort remain obesity, waist circumference, and lifestyle factors [35, 36].

The cardiovascular consequences of pediatric hypertension represent an alarming and preventable health threat [4]. In our grade 9 cohort, echocardiographic measures revealed a significant increase in left ventricular posterior wall thickness (LVPWd) among hypertensive adolescents compared to their pre-hypertensive peers with P value 0.012. These findings suggest early target-organ damage, particularly left ventricular hypertrophy (LVH), a well-established predictor of future cardiovascular morbidity and mortality [4]. The detection of such changes at this young age highlights a substantial lifetime burden, as persistent hypertension can accelerate atherosclerosis, impair cardiac function, and predispose arrhythmias [37]. Similar observations were reported by Urbina and his coworkers [38], who documented increased left ventricular mass and wall thickness in hypertensive adolescents, and by Litwin and his collages, who demonstrated that pre-hypertension is associated with subtle cardiac structural changes. The concordance of our findings with the broader literature reinforces the urgent need for early screening, diagnosis, and intervention to mitigate the long-term cardiovascular impact of pediatric hypertension [39, 40].

## Conclusion

Ambulatory blood pressure monitoring (ABPM) is a valuable tool for accurately diagnosing hypertension in children and adolescents, highlighting the role of obesity and lifestyle factors in elevated blood pressure. The study underscores the need for early interventions, including healthy diet and weight management, to prevent long-term cardiovascular risks. It also draws attention to the dual challenges of undernutrition and overnutrition, rising hypertension rates, and the importance of promoting balanced nutrition, physical activity, and health education. School, community, and policy-level interventions are essential to protect future generations’ health.

## Limitations

This study has several limitations. It was conducted in a single private school serving primarily middle-class students, which may limit the generalizability of findings to other socio-economic groups across Egypt. The sample size was relatively small, and sex-based differences were not fully examined, highlighting the need for future sex-specific studies. Although follow-up is ongoing, long-term data are not yet available. Parent response rates were low, likely reflecting reluctance or limited awareness about childhood hypertension, which underscores broader gaps in community knowledge. Future studies should include a more diverse range of schools and larger cohorts to better capture national trends and validate these findings.

## Data Availability

The datasets generated and analyzed during the current study are available from the corresponding author upon reasonable request.
